# Dose‐dependent induction of epithelial‐mesenchymal transition in 3D melanoma models by non‐thermal plasma treatment

**DOI:** 10.1002/1878-0261.70055

**Published:** 2025-05-29

**Authors:** Eline Biscop, Edgar Cardenas De La Hoz, Hanne Verswyvel, Joey De Backer, Ho Wa Lau, Angela Privat‐Maldonado, Wim Vanden Berghe, Steve Vanlanduit, Evelien Smits, Annemie Bogaerts, Abraham Lin

**Affiliations:** ^1^ PLASMANT, Department of Chemistry University of Antwerp Belgium; ^2^ Center for Oncological Research – Integrated Personalized & Precision Oncology Network (IPPON) University of Antwerp Belgium; ^3^ Industrial Vision Lab, Department of Applied Engineering University of Antwerp Belgium; ^4^ Cell Death Signaling Epigenetics Lab – Integrated Personalized & Precision Oncology Network (IPPON University of Antwerp) Belgium

**Keywords:** dielectric barrier discharge, epithelial‐mesenchymal transition, melanoma, plasma medicine, plasma oncology

## Abstract

Despite the promising results of non‐thermal plasma (NTP) devices for cancer therapy, the potential adverse effects of NTP irradiation have remained unexplored, including the effects on epithelial‐mesenchymal transition (EMT) and subsequent cancer metastasis. In this study, we investigate NTP‐induced EMT initiation and progression. A microsecond‐pulsed dielectric barrier discharge plasma device was used for NTP treatment, and chicken chorioallantoic membrane (CAM) and spheroids were used as 3D tumor models. NTP treatment reduced tumor volume in the CAM model, but a shift towards a mesenchymal‐like phenotype was also measured in melanoma tumors via analysis of the six EMT biomarkers, though changes in cancer cell migration to other organs were not significant. In the spheroid model, molecular analysis also indicated an EMT response following NTP treatment, and enhanced cell migration was measured in one cell line. EMT induction with NTP was dose‐dependent and transient; high NTP treatments caused significant EMT response and enhanced migration, but low NTP doses did not. Our findings highlight the important role of NTP parameters for cancer treatment and consequential EMT responses. The insights obtained here further build the foundation for clinical optimization, harnessing the cancer‐killing potential of NTP while safeguarding against undesirable EMT‐related outcomes.

## Graphical abstract

Non‐thermal plasma treatment of melanoma cells induced epithelial‐mesenchymal transition (EMT) in a dose‐dependent fashion. This report highlights the critical need to further investigate potential adverse effects of non‐thermal plasma for cancer therapy and to optimize treatment parameters for clinical translation.

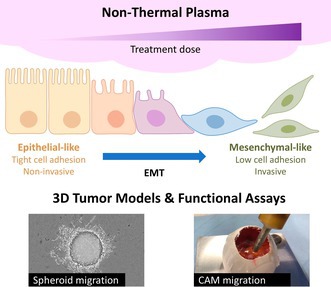

**Abbreviations**
CAM: chicken chorioallantoic membrane
ED: embryonic development
EMT: epithelial‐mesenchymal transition
FBS: fetal bovine serum
FE‐DBD: floating‐electrode dielectric barrier discharge
GFR: growth factor‐reduced
NTP: non‐thermal plasma
PFA: paraformaldehyde
RONS: reactive oxygen and nitrogen species
SEM: standard error of the mean
TBS: Tris buffer saline

## 1 Introduction

Non‐thermal plasma (NTP) is a partially ionized gas generated at atmospheric conditions and consisting of various reactive oxygen and nitrogen species (RONS) [1, 2]. Due to the capacity to locally generate and control the delivery of short‐lived RONS (e.g., ^·^OH, ^·^NO, O) to biological substrates, NTP has gained a lot of interest for several biomedical applications, with a growing interest as an alternative or additional therapy for cancer [1, 2, 3, 4]. Over the past three decades, studies have demonstrated very promising results in killing cancer cells with NTP, using *in vitro* models, where efficacy is demonstrated in multiple cancer types. In animal models, NTP reduced tumor burden and increased survival in mice, and in pilot clinical studies, no significant adverse side effects were documented [5, 6, 7, 8, 9, 10, 11, 12, 13, 14].

While NTP exposes biological targets to various physical components (e.g., UV, high electric/magnetic fields), numerous studies have shown that anti‐cancer effects are predominantly due to the formation of highly reactive RONS, mainly H_2_O_2_, ${\mathrm{NO}}_{2}^{-}$, ${\mathrm{NO}}_{3}^{-}$, ^·^OH, ^1^O_2_, O/O_3_, and ^·^NO, which can elicit oxidative stress responses [15, 16].

Although the anti‐cancer potential of NTP through the induction of oxidative stress appears promising, extensive research in the field of redox biology, from both experimental and clinical studies, indicates that RONS can also play an important role in promoting various stages of tumor formation and progression. This phenomenon is dependent on their mutagenic potential and ability to interact with signaling pathways that regulate cell proliferation, motility, invasiveness, and survival [17, 18]. Increasing evidence suggests that oxidative stress plays a crucial role in promoting cell invasion and metastasis through the activation of stimuli associated with epithelial‐mesenchymal transition (EMT) [17], a morphogenesis process first described by researchers studying embryonic and organ development [19]. This duality of EMT to play critical roles in tissue regeneration and cancer metastasis led to a continuous surge in research attention [19]. In the context of cancer, EMT represents a transition from an epithelial phenotype towards a mesenchymal phenotype during which the cancer cells lose their apical‐basal polarity and acquire more migratory and invasive properties [19, 20]. As implied by its name, EMT is a transition process wherein cells go through different states along the E‐M spectrum before attaining a fully mesenchymal phenotype, also called EMT plasticity [21, 22, 23]. Consequently, this generates an extensive phenotypic heterogeneity within the tumor, resulting in increased adaptability and resistance to therapies, and granting cancer cells a survival advantage [24, 25].

Given the established connection between oxidative stress and EMT, we aimed to investigate whether NTP would initiate this transition. To better fill this gap in fundamental understanding, we performed a comprehensive screen of six key EMT biomarkers and evaluated the resulting change in the cell migratory capacity post‐NTP treatment. We hypothesized that altering NTP treatment parameters to control the delivery of RONS would modulate induced oxidative stress and subsequent EMT response. In this study, we investigated the NTP effects on EMT in melanoma cells (A375, SK‐MEL‐28, and Malme‐3M), as melanomas are the most accessible cancers for direct clinical treatment with medical NTP devices [26, 27, 28]. Furthermore, we evaluated EMT responses using two 3D cancer models: the 3D *in vitro* spheroid model and the chicken chorioallantoic membrane (CAM) *in ovo* model. Traditional 2D cell monolayers are severely limited in representing the tumor environment, whereas the 3D models used here better recapitulate essential tumorigenic phenotypes including multi‐dimensional cellular interactions, diffusion gradient of oxygen and nutrients, spontaneous development of hypoxic regions, angiogenesis, and even invasive and metastatic capacity [29, 30, 31, 32]. Our results on these more representative tumor models elucidated the nature of the NTP‐induced EMT response. In the CAM model, 700 Hz NTP treatment induced significant anti‐cancer effects, as evidenced by reduced tumor weight. At this treatment, EMT biomarkers in the tumor indicated reduced epithelial expression and more mesenchymal expression. Both low‐(3.52 J) and high‐(12.3 J) dose NTP treatment reduced spheroid size, but only the high‐dose treatment induced a dynamic shift towards mesenchymal marker expression. Whereas high NTP treatments clearly initiate EMT, low NTP treatments triggered no such response. Therefore, not only does this study provide crucial insight into a fundamental gap in knowledge concerning NTP‐cell interactions, but our findings also highlight the importance of optimizing NTP treatment parameters as a balance between induced anti‐cancer effects and EMT responses. As clinical translation of NTP technology moves forward, it is crucial to consider the duality of NTP‐induced oxidative stress, and this study defines potential boundaries for further clinical refinement.

## 2 Materials and methods

### 2.1 Cell culture

For this study, three human melanoma cell lines were used: A375 (ATCC, CRL‐1619TM; RRID: CVCL_0132), SK‐MEL‐28 (ATCC, HTB‐72; RRID: CVCL_0526) and Malme‐3M (ATCC, HTB‐64; RRID: CVCL_1438). All cell lines were cultured in Dulbecco's Modified Eagle Medium (DMEM) (Gibco™, Life Technologies, Waltham, MA, USA, 10938‐025) supplemented with 10% Fetal Bovine Serum (FBS) (Gibco™, Life Technologies, 10270‐106), 100 units·mL^−1^ penicillin/streptomycin (Gibco™, Life Technologies, 15140‐122) and 4 mm l‐glutamine (Gibco™, Life Technologies, 25030‐024). The cells were maintained at 37 °C in a humidified atmosphere with 5% CO_2_. All cell lines have been authenticated using STR profiling every year, and all experiments were performed with mycoplasma‐free cells.

### 2.2 3D spheroid model

For the generation of 3D spheroids, the cells were seeded in an ultra‐low attachment 96‐well plate (Corning B.V. Life Sciences, Amsterdam, NL, 7007). Spheroids were made with 5000 cells per well for SK‐MEL‐28 and 7000 cells per well for Malme‐3M and supplemented with 2% GFR Matrigel (Corning B.V. Life Sciences, 354‐230). Different seeding concentrations were used for the different cell lines in order to obtain spheroids with a diameter of approximately 500 μm at the start of the experiments. For the A375 cell line, no spheroids could be generated due to the incapability of this cell line to form compact spheres. After seeding, the spheroids were centrifuged at 200 ***g*** for 10 min at 4 °C. Subsequently, they were incubated for 3 days at 37 °C in a humidified incubator with 5% CO_2_ prior to their use in experiments. For each independent experiment, five spheroids were generated and allocated per experimental condition. These five were used to compensate for potential loss of spheroids during handling and transferring so that a minimum of three spheroids per experimental condition was analyzed.

### 2.3 3D CAM in ovo model

Fertilized leghorn chicken eggs (Kwekerij Wyverkens, Halle, BE) (embryonic development (ED) day 4) were incubated in a horizontal position at 37 °C and 65% humidity in an egg incubator with automatic turning function (Ova‐Easy 100, Brinsea, Veenendaal, NL). On ED day 5, the upper pole of the egg was disinfected and pierced with a 20G sterile needle (BD, Franklin Lakes, NJ, USA) after which it was sealed with medical tape (Leukosilk S, Covamed Farma BVBA, Marke, BE). Following the piercing, the eggs were put in a vertical position in the egg incubator (turning function off) to promote the relocation of the air chamber to the upper pole. At ED day 7, the upper pole of the egg was cut off to expose the CAM, and a 1 × 1 mm filter paper soaked in diethyl ether was briefly applied on a vascular region on the CAM. At this region, a sterile silicon ring (ID = 5 mm, OD = 6 mm) was placed on the CAM and 2 × 10^6^ cells for A375 and SK‐MEL‐28 or 6 × 10^6^ cells for Malme‐3M, mixed with 15 μL growth factor‐reduced (GFR) Matrigel, were loaded into the ring. The eggs were sealed with Tegaderm (3D) and placed back in the incubator for 4 days. On ED day 11, the Tegaderm was cut and the tumors were treated with either direct NTP or an EMT‐inducer. For the EMT‐inducer, a second sterile plastic ring (ID = 7 mm, OD = 8.5 mm) was placed around the first ring and 20 μL of StemXVivo EMT Inducing Media Supplement (100×) (Biotechne, Minneapolis, MN, USA, CCM017) was loaded into the ring. This EMT‐supplement contains the following premium quality proteins and high performance neutralizing antibodies to induce EMT: Recombinant Human Wnt‐5a, Recombinant Human TGF‐β1, Anti‐Human E‐cadherin, Anti‐Human sFRP‐1, and Anti‐Human Dkk‐1. The eggs were sealed with Tegaderm and incubated until the end of the experiment. On ED day 14, the tumors were excised and weighed on a precision balance (Mettler Toledo, Fischer, Merelbeke, BE) after which the chicken embryos were further dissected to collect the liver, lungs, and a lower part of the CAM for further analysis. All steps outside the incubator were carried out using a heat block (set at 37 °C) with a custom‐made egg‐shaped aluminum adapter. For each independent experiment, seven eggs were allocated per experimental condition. Not every egg successfully developed a tumor, and in general, five tumors were collected per condition for analysis at the end of the experiment. The experiments presented here were terminated on day 14 of chick embryo development, and thus ethical review and approval were waived for this study, as according to European regulations, only experiments on chick embryos terminated after day 15 of development need to be declared.

### 2.4 NTP treatment

**Fig. 1:**
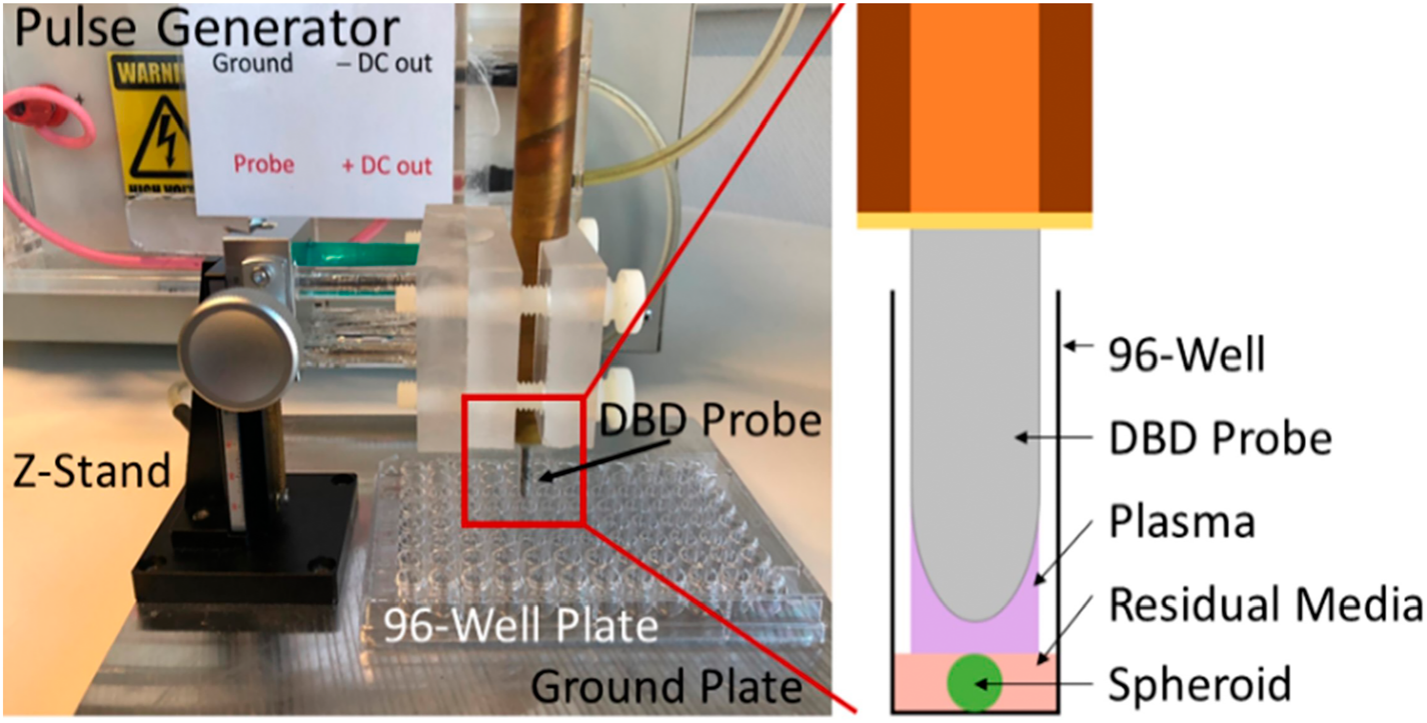
The microsecond‐pulsed floating‐electrode dielectric barrier discharge (FE‐DBD) plasma system used in this study for non‐thermal plasma (NTP) treatment. Spheroids were transferred to an empty 96‐well plate in 3 μL of medium for NTP treatment.

We used a microsecond‐pulsed floating‐electrode dielectric barrier discharge (FE‐DBD) plasma system, described previously in detail [33, 34], to generate NTP with a 30 kV pulse, 1–1.5 μs rise time, and 2 μs pulse width. For the treatment of spheroids, each spheroid is transferred to an empty 96‐well plate in 3 μL of medium prior to treatment (Fig. 1). The gap between the electrode and the spheroid was fixed at a 1 mm distance using a z‐positioner, following which NTP was discharged directly on the cells for 10 s at various frequencies, ranging between 100 and 700 Hz. The energy per pulse of each NTP discharge was previously measured and reported (1.76 ± 1.15 mJ per puse) [33, 34], and therefore, the total treatment energy dose ranged from 1.76 to 12.3 J. Since NTP treatment frequency was the only variable parameter and we aimed to hold all other parameters constant, we displayed NTP treatment dose as frequency. Moreover, it should also be noted that while we aimed to hold application distance constant, this was much more difficult for 3D spheroids and CAM models compared to 2D cell monolayers, which may influence the total treatment energy dose. Thus we believe the most accurate representation of our NTP treatment dose is to provide, in detail, the parameters used for treatment, though to be concise, it was reported as treatment frequency in the Results section.

Immediately following treatment, 100 μL of fresh cell culture medium was replenished in the well. For the treatment of the *in ovo* tumors, the gap between the electrode and the tumor was set at approximately 2–4 mm distance. Fixing at an exact distance was not possible for this model due to the varying egg size and occasional movement of the embryo. NTP was discharged directly onto the tumor for 10 s at 700 Hz, following which the eggs were sealed again. As a positive control for EMT induction, the StemXVivo EMT Inducing Media Supplement, later referred to as EMT‐supplement, was used.

### 2.5 Immunofluorescence on 3D spheroids and in ovo tumors

To assess several key EMT biomarkers, immunofluorescent staining was performed on both 3D spheroids and tumors collected from the *in ovo* model. The spheroids were collected 1, 4, and 7 days after NTP treatment and fixed overnight in 4% paraformaldehyde (PFA) at 4 °C. The fixed spheroids were transferred to multiarray molds of 4% agarose, following which they were dehydrated and embedded in paraffin overnight. *In ovo* tumors were collected 3 days after NTP treatment and fixed with 4% PFA at room temperature prior to dehydration and paraffin embedding. For both embedded spheroids and *in ovo* tumors, sections of 5 μm were cut. The slides were deparaffinized and rehydrated, following which antigen retrieval was done using a citrate buffer (10 mm, pH 6) for 20 min at 96 °C. After a 30 min cooldown, the slides were washed twice in Tris buffer saline with 2.5% Triton X‐100 (TBS) for 5 min. Subsequently, the slides were blocked for 2 h with TBS containing 10% goat serum (Abcam, Cambridge, UK, ab7481) for vimentin and ZEB1 stainings, or with 10% donkey serum (Abcam, ab7475) for E‐cadherin, N‐cadherin, SNA1, and Twist staining. After blocking, the primary antibody was applied, diluted in TBS containing 1% goat or donkey serum, depending on the antibody, and incubated overnight at 4 °C. The following primary antibodies were used: E‐cadherin (Abcam, ab76055, 1 : 200), N‐cadherin (Thermo Fisher, Waltham, MA, USA, MA5‐15633, 1 : 200), Vimentin (Abcam, ab92547, 1 : 500), SNAI1 (Santa Cruz Biotechnology, Dallas, TX, USA, sc‐271977, 1 : 100), ZEB1 (Abcam, ab203829, 1 : 250) and Twist (Abcam, ab175430, 1 : 200). The next day, the slides were washed twice in TBS and incubated with the secondary antibody in the dark for 1 h at room temperature. The following secondary antibodies were used: Goat Anti‐Rabbit IgG (H + L) Alexa Fluor 488 (Abcam, ab150077) and Donkey Anti‐Mouse IgG (H + L) Highly Cross‐adsorbed Alexa Fluor 594 (Thermo Fisher, A21203). The slides for vimentin and ZEB1 were additionally chemically bleached with Sudan Black B in 70% ethanol for 30 min to reduce autofluorescence, following which the slides were washed three times in TBS. All slides were mounted with VECTASHIELD HardSet antifade mounting medium with DAPI (Vectorlabs, Burlingame, CA, USA). Mean fluorescence intensity was analyzed using the imagej software (National Institute of Health, Bethesda, MD, USA). Both DAPI and Alexa Fluor 488 or Alexa Fluor 594 were measured, depending on the secondary antibody used. DAPI staining was used to normalize the fluorescence intensity for each slide. With regard to *in ovo* sections, a distinction was made between the chicken cells and the human cells, and analysis was only performed on the human cells. With regard to spheroids, several spheroids were collected and sectioned in one single paraffin block, and the entire spheroid section was analyzed. Since each spheroid was only 500 μm in diameter, it was not possible to section at the same depth. Instead, 20 spheroid sections were collected from the paraffin blocks, and the largest spheroid sections were used for staining to also include the deeper regions of the spheroid [35].

### 2.6 Spheroid migration

In order to evaluate cell migration in a more functional manner, a spheroid migration assay was performed. In this assay, the treated spheroids were immediately transferred to a collagen‐coated 96‐well plate in 300 μL growth factor‐reduced medium (2% FBS instead of 10% FBS). The spheroids were incubated for 30 min to allow them to adhere to the coated surface before starting the imaging. To investigate cell migration out of the spheroids, images were collected every 4 h for a duration of 3 days using the Tecan Spark Cyto 600 (Tecan, Männedorf, Switzerland). These images were subsequently analyzed using the orbits oncology software (Orbits Oncology Inc, Palo Alto, CA, USA) [35, 36].

### 2.7 RNA isolation and cDNA conversion

To isolate the RNA from the liver, lungs, and lower CAM of the chicken embryos, the RNeasy Plus Mini Kit (Qiagen, Hilden, DE, Germany, 74034) was used according to the manufacturer's instructions. The RNA was then quantified using the Qubit 4 Fluorometer following which cDNA conversion was performed using Superscript II Reverse Transcriptase (Thermo Fisher, 18061‐022) according to the manufacturer's instructions.

### 2.8 Real‐time PCR

Amplification of cDNA was performed using the GoTaq Flexi DNA Polymerase assay (Promega, Madison, WI, USA, M829B) with the Techne Prime Thermal Cycler. The following conditions were used during PCR: 95 °C for 2 min, 35 cycles of: 95 °C for 0.5 min, 60 °C for 0.5 min, and 72 °C for 1 min; 72 °C for 5 min. The following candidate reference genes were assessed: B2M, HMBS, ALTB, TBP, SDHA, YQHAZ, and UBC. The reference gene β‐2 microglobulin (B2M, GeneID: 567) was used to selectively prime human cDNA. Relative quantification was performed using imagej software.

### 2.9 Statistical analysis

For each assay, three independent experiments were performed with at least 3–7 independent replicates, subject to model or handling complications. All statistical differences were analyzed using the linear mixed model with jmp pro 13 (SAS Software, Cary, NC, USA) in order to test for fixed effects while accounting for random effects and interactions due to biological and experimental variability. When a significant difference was detected, the *post hoc* Dunnett's test was performed to calculate the adjusted *P* value compared to the control. A *P* value of < 0.05 was considered statistically significant. Data in all graphs are represented as mean ± standard error of the mean (SEM); the number of independent experiments and replicates are indicated in the legend, and all figures were prepared in graphpad prism (GraphPad Prism 10.0.1, GraphPad Prism Software, Inc., Boston, MA, USA).

## 3 Results

### 3.1 Investigation of key EMT biomarkers after NTP treatment in the 3D CAM *in ovo* model

**Fig. 2:**
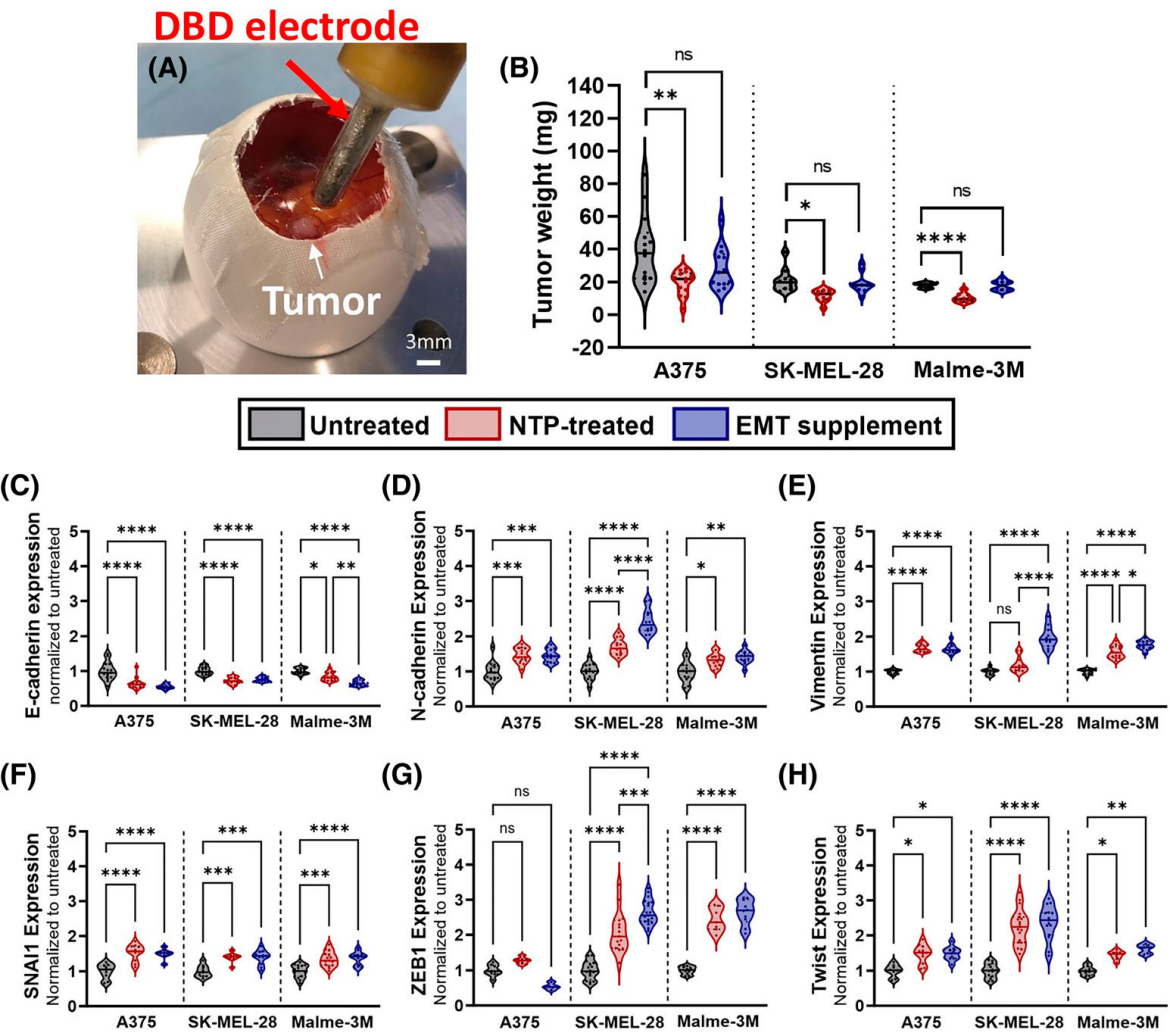
Non‐thermal plasma (NTP)‐induced epithelial‐mesenchymal transition (EMT) response in tumors collected from the chicken chorioallantoic membrane (CAM) *in ovo* model. (A) Image showing NTP treatment of the tumor grafted on top of the CAM. Scale bar is 3 mm. (B) NTP treatment at 700 Hz induced anti‐cancer response as seen in the reduction in tumor weight. (C–H) Results from immunofluorescent staining of six key EMT biomarkers ((C) E‐cadherin, (D) N‐cadherin, (E) Vimentin, (F) SNAI1, (G) Twist, and (H) ZEB1) in tumor sections collected from the CAM *in ovo* model. An EMT‐inducing supplement was used as a positive control. The results are normalized to the untreated sample of each melanoma cell line. The data are represented as mean ± SEM of three independent experiments with 3–5 replicates per experiment, as represented by each individual data point in the graph. (C–H) Data on all graphs are normalized to untreated controls (*y*‐axis). DBD, dielectric barrier discharge; EMT, epithelial‐mesenchymal transition; NTP, non‐thermal plasma. Statistical significance was calculated using the generalized linear mixed model. *P ≤ 0.05, **P ≤ 0.01, ***P ≤ 0.001, ****P ≤ 0.0001.

EMT is a complex multifaceted cellular process involving changes in cellular protein expression and physiology, resulting in the loss of epithelial characteristics and gain of mesenchymal traits [17]. In order to evaluate the initial impact of NTP on EMT progression, we assessed the expression of key EMT biomarkers following NTP treatment in the 3D chicken CAM *in ovo* model. This model provides an *in vivo*‐like environment that closely resembles physiological conditions (e.g., extracellular matrix, 3D cell interaction, angiogenesis, hypoxic regions). These features make it an excellent preclinical model to study cancer metastasis in a considerably shorter time compared to murine models (14 days instead of 4–10 weeks) [37]. Solid tumors are grown on top of the CAM (Fig. 2A), following which they are treated with NTP (700 Hz, 10 s). Three days after treatment, tumors were collected, weighed, fixed, and embedded in paraffin for sectioning. The 700 Hz NTP treatment induced a cytotoxic response in all three melanoma cell lines, as evidenced by the significant reduction in tumor weight when compared to the untreated tumors (Fig. 2B). Immunofluorescence staining of tumor sections was used to examine the expression of the EMT biomarkers. One of the most important hallmarks of EMT is the so‐called ‘cadherin switch’, which is represented by downregulation of E‐cadherin and upregulation of N‐cadherin on the cell membrane surface [22, 38]. E‐cadherin is one of the most important molecules regulating cell–cell adhesion in epithelial tissues, and N‐cadherin endows tumor cells with enhanced migratory and invasive capacity. Our results showed that NTP treatment downregulates E‐cadherin expression in all three cell lines: A375 (0.64 ± 0.04‐fold change, *P* ≤ 0.0001), SK‐MEL‐28 (0.72 ± 0.02‐fold change, *P* ≤ 0.0001) and Malme‐3M (0.83 ± 0.03, *P* = 0.0121) (Fig. 2C). In contrast, N‐cadherin expression is upregulated in all three cell lines: A375 (1.46 ± 0.06‐fold change, *P* = 0.0002), SK‐MEL‐28 (1.71 ± 0.06‐fold change, *P* ≤ 0.0001) and Malme‐3M (1.32 ± 0.05‐fold change, *P* = 0.0250) (Fig. 2D). This dynamic transition is in line with the positive control, an EMT‐inducing supplement, indicating that NTP is indeed promoting EMT.

Although the cadherin switch is an important hallmark of EMT, this alone is not sufficient to trigger EMT‐associated changes in cellular motility, and modulation of other markers is needed. Vimentin, a type III intermediate filament predominantly found in mesenchymal cells, has also shown to be an excellent indicator for EMT, as it is known to maintain cellular integrity and provide resistance against mechanical stress via a viscoelastic framework [20, 22]. Hence, increased expression of vimentin promotes cancer cell migration [20]. The analysis of vimentin expression shows an upregulation for A375 (1.68 ± 0.04‐fold change, *P* ≤ 0.0001) and Malme‐3M (1.56 ± 0.05‐fold change, *P* ≤ 0.0001), but not for SK‐MEL‐28, although an increasing trend was noted (Fig. 2E). This behavior is also in line with the results obtained using the EMT‐supplement, further supporting the hypothesis that NTP promotes EMT.

Lastly, we investigated several transcription factors that regulate EMT, namely from the Snail, ZEB, and Twist families [39]. Altogether they act to suppress the expression of epithelial markers such as E‐cadherin, as well as activate mesenchymal markers among which are N‐cadherin and vimentin. In general, Snail, also known as SNAI1, is primarily known as a major inducer of EMT by directly regulating the expression of E‐cadherin and vimentin and also stimulating the activation of Twist and ZEB1, which are involved in upholding the mesenchymal phenotype [19]. With regard to SNAI1, we observed an increase in expression for all three cell lines: A375 (1.53 ± 0.07‐fold change, *P* ≤ 0.0001), SK‐MEL‐28 (1.40 ± 0.04‐fold change, *P* = 0.0007), and Malme‐3M (1.35 ± 0.05‐fold change, *P* = 0.0002). This expression is comparable to that of the EMT‐inducing supplement (Fig. 2F). Furthermore, while A375 tumors only showed an upregulation for Twist expression (1.47 ± 0.08‐fold change, *P* = 0.0254), both Twist and ZEB expression are upregulated for SK‐MEL‐28 tumors (Twist: 2.3 ± 0.1‐fold change, *P* ≤ 0.0001; ZEB1: 2.0 ± 0.1‐fold change, *P* ≤ 0.0001) and Malme‐3M tumors (Twist: 1.40 ± 0.05‐fold change, *P* = 0.0144; ZEB1: 2.4 ± 0.1‐fold change, *P* ≤ 0.0001) (Fig. 2G,H). Since these transcription factors are often found to be co‐expressed in different combinations during EMT, the absence of one of them does not necessarily imply that EMT does not occur [22, 40].

Taken together, these findings suggest that NTP treatment triggers an EMT response, as evidenced by the activation of the cadherin switch, the upregulation of vimentin, and the activation of the three main EMT transcription factors. Nevertheless, it should be noted that these findings only represent one time point after NTP treatment, whereas EMT is a dynamic process that transitions through different hybrid phases before attaining a fully mesenchymal phenotype. During these hybrid phases, the cells can also recover and return to their initial epithelial state before becoming fully mesenchymal [21]. To further investigate the progression of the EMT process, we looked at later time points using the 3D spheroid model, as the CAM model is restricted to a 14‐day limit due to ethical considerations.

### 3.2 Investigation of EMT progression and NTP dose dependence in the 3D spheroid model

**Fig. 3:**
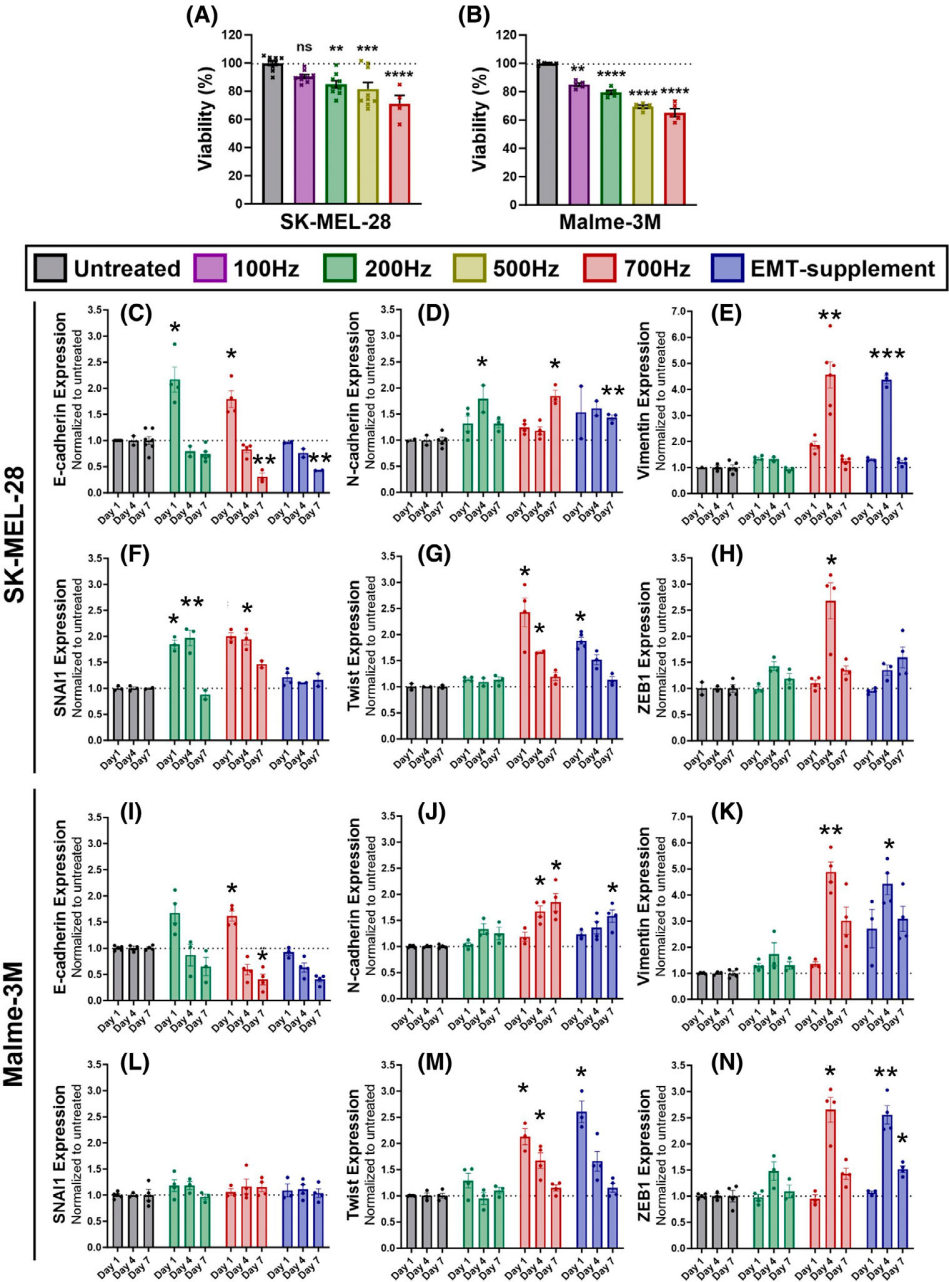
Non‐thermal plasma (NTP)‐induced epithelial‐mesenchymal transition (EMT) response in 3D melanoma spheroids. (A) Cytotoxic response of different NTP doses on SK‐MEL‐28 spheroids ranging from 100 Hz (1.76 J) to 700 Hz (12.3 J). (B) Cytotoxic response of different NTP doses on Malme‐3M spheroids. (C–H) Results from immunofluorescent staining of six key EMT biomarkers ((C) E‐cadherin, (D) N‐cadherin, (E) Vimentin, (F) SNAI1, (G) Twist, and (H) ZEB1) in SK‐MEL‐28 spheroids comparing a low dose (200 Hz) and high dose (700 Hz) NTP treatment. As a positive control, an EMT‐inducing supplement was used. (I–N) Results from immunofluorescent staining of six key EMT biomarkers ((I) E‐cadherin, (J) N‐cadherin, (K) Vimentin, (L) SNAI1, (M) Twist, and (N) ZEB1) in Malme‐3M spheroids. The results from (C–N) were normalized to the untreated sample of the same time point, and alterations in expression are assessed by calculating statistical significance between the treated sample and the untreated sample from the same time point. The untreated samples weree used to indicate the baseline expression at each time point. Data are represented as mean ± SEM of three independent experiments with 3–5 replicates per experiment. (C–N) Data on all graphs are normalized to untreated controls (*y*‐axis) for day 1, 4, and 7 (*x*‐axis). EMT, epithelial‐mesenchymal transition. Statistical significance between the treated samples and the untreated samples from the same time point was calculated using the generalized linear mixed model. *P ≤ 0.05, **P ≤ 0.01, ***P ≤ 0.001.

To examine the further progression of EMT, we evaluated the expression of the six biomarkers at day 1, 4, and 7 after NTP treatment of melanoma spheroids. For these experiments, only SK‐MEL‐28 and Malme‐3M cell lines were used, since A375 cells did not form tight spheroids. NTP treatment was able to significantly reduce spheroid viability, both at a low‐dose NTP treatment (200 Hz, 3.52 J) and a high‐dose NTP treatment (700 Hz, 12.3 J) (Fig. 3A,B).

Based on the cadherin analysis, interestingly, there was an initial spike in E‐cadherin expression on day 1 for both NTP treatments of the SK‐MEL‐28 spheroids (200 Hz treatment: 2.2 ± 0.2‐fold change, *P* = 0.0481; 700 Hz treatment: 1.8 ± 0.2‐fold change, *P* = 0.0469) (Fig. 3C), and for the 700 Hz treatment of the Malme‐3M spheroids (1.6 ± 0.1‐fold change, *P* = 0.0193) (Fig. 3I), indicating an increase in cell adhesion. However, by day 4, the expression diminished and aligned with that of the untreated sample. By day 7, a strong downregulation of E‐cadherin was observed for both cell lines for the high‐dose NTP treatment (0.31 ± 0.07 for SK‐MEL‐28, *P* = 0.0013, and 0.4 ± 0.1 for Malme‐3M, *P* = 0.0241); whereas, despite the declining trend, no significant downregulation of E‐cadherin was observed for the low NTP dose.

N‐cadherin expression was upregulated for the high‐dose NTP treatment by day 7 for SK‐MEL‐28 spheroids (1.8 ± 0.1‐fold change, *P* = 0.0156) (Fig. 3D) and for Malme‐3M spheroids (1.8 ± 0.2‐fold change, *P* = 0.0440) (Fig. 3J). However, the low‐dose NTP treatment did not significantly alter the expression of N‐cadherin.

Only the high‐dose NTP treatment induced an increase in vimentin expression by day 4 for both cell lines (4.6 ± 0.5‐fold change for SK‐MEL‐28, *P* = 0.0029; 4.9 ± 0.4‐fold change for Malme‐3M, *P* = 0.0059) (Fig. 3E,K). Interestingly, the expression decreased again by day 7 and, for SK‐MEL‐28, even completely returned to the baseline level. Similar to the cadherin switch, the low‐dose NTP treatment had a negligible impact on vimentin expression.

Lastly, we examined the expression of the three EMT transcription factors. The expression of SNAI1 already increased 1 day post‐NTP treatment for the SK‐MEL‐28 spheroids, both at low‐dose NTP treatment (1.85 ± 0.08‐fold change, *P* = 0.0127) and high‐dose NTP treatment (2.00 ± 0.07, *P* = 0.0061), which slightly decreased again by day 7 (Fig. 3F). However, neither NTP treatment had an impact on SNAI1 expression in the Malme‐3M spheroids (Fig. 3L). For Twist and ZEB1, the two cell lines respond similarly to NTP treatment. More specifically, only the high‐dose NTP treatment affected Twist expression, with the highest upregulation at day 1 (2.4 ± 0.3‐fold change for SK‐MEL‐28, *P* = 0.0481 and 2.1 ± 0.2 for Malme‐3M, *P* = 0.0373), followed by a gradual return to baseline levels by day 7 (Fig. 3G,M). Furthermore, ZEB1 expression was upregulated by day 4 following the high‐dose NTP treatment of both spheroids (2.7 ± 0.3‐fold change for SK‐MEL‐28, *P* = 0.0478, and 2.7 ± 0.2‐fold change for Malme‐3M, *P* = 0.0159) (Fig. 3H,N). Similar to Twist, this effect was reduced back to its baseline level by day 7.

Altogether, these results suggest that the high‐dose NTP treatment triggered an EMT response, as evidenced by the activation of the cadherin switch, the upregulation of vimentin, and the activation of the three main EMT transcription factors. However, these responses seem to be temporary, and most of the biomarkers we investigated returned to their baseline levels by day 7, denoting a partial EMT instead of reaching a fully mesenchymal phenotype. In contrast, while the low‐dose NTP treatment induced a cytotoxic response, it did not trigger a response for most EMT biomarkers, suggesting that using lower NTP treatment doses could circumvent these issues and be more attractive from a clinical perspective.

### 3.3 Functional evaluation of EMT properties post‐NTP treatment in both 3D melanoma models

Whereas the expression of key EMT biomarkers is a valuable indicator of EMT progression, defining EMT exclusively on the basis of molecular markers underrepresents the enormous complexity and plasticity of EMT [22]. To complement this data, we performed functional assays to investigate whether NTP‐induced EMT could enhance the migratory capacity of cancer cells. In the CAM *in ovo* model, we collected several organs from the chicken embryo to check for metastasis of the tumor from the CAM after treatment. The liver, lungs, and lowest part of the CAM (furthest away from the tumor site) were collected immediately after tumor removal, and RNA was isolated. Using RT‐PCR, the presence of human RNA in the organs of the chicken embryo was investigated to identify whether the human melanoma cells had metastases from the primary tumor. Organs from an egg without a tumor were used to test eight primers for cross‐reactivity with the chicken cells, namely B2M, HMBS, ALTB, TBP, SDHA, YWHAZ, and UBC. From these, only B2M, TBP, and HMBS gave a positive result for the human control sample without showing any reactivity with chicken samples (Table 1). Since B2M gave the brightest signal, we continued with this primer.

**Table 1:** Cross‐reactivity test PCR primers.

	Chicken samples						Positive control
	CAM1	CAM2	Liver1	Liver2	Lung1	Lung2	A375
B2M	−	−	−	−	−	−	++
HMBS	−	−	−	−	−	−	+
ALTB	−	−	−	+	−	+	++
TBP	−	−	−	−	−	−	+
SDHA	−	−	−	−	−	−	−
YWHAZ	−	−	−	−	−	−	−
UBC	−	−	−	−	−	−	−

**Fig. 4:**
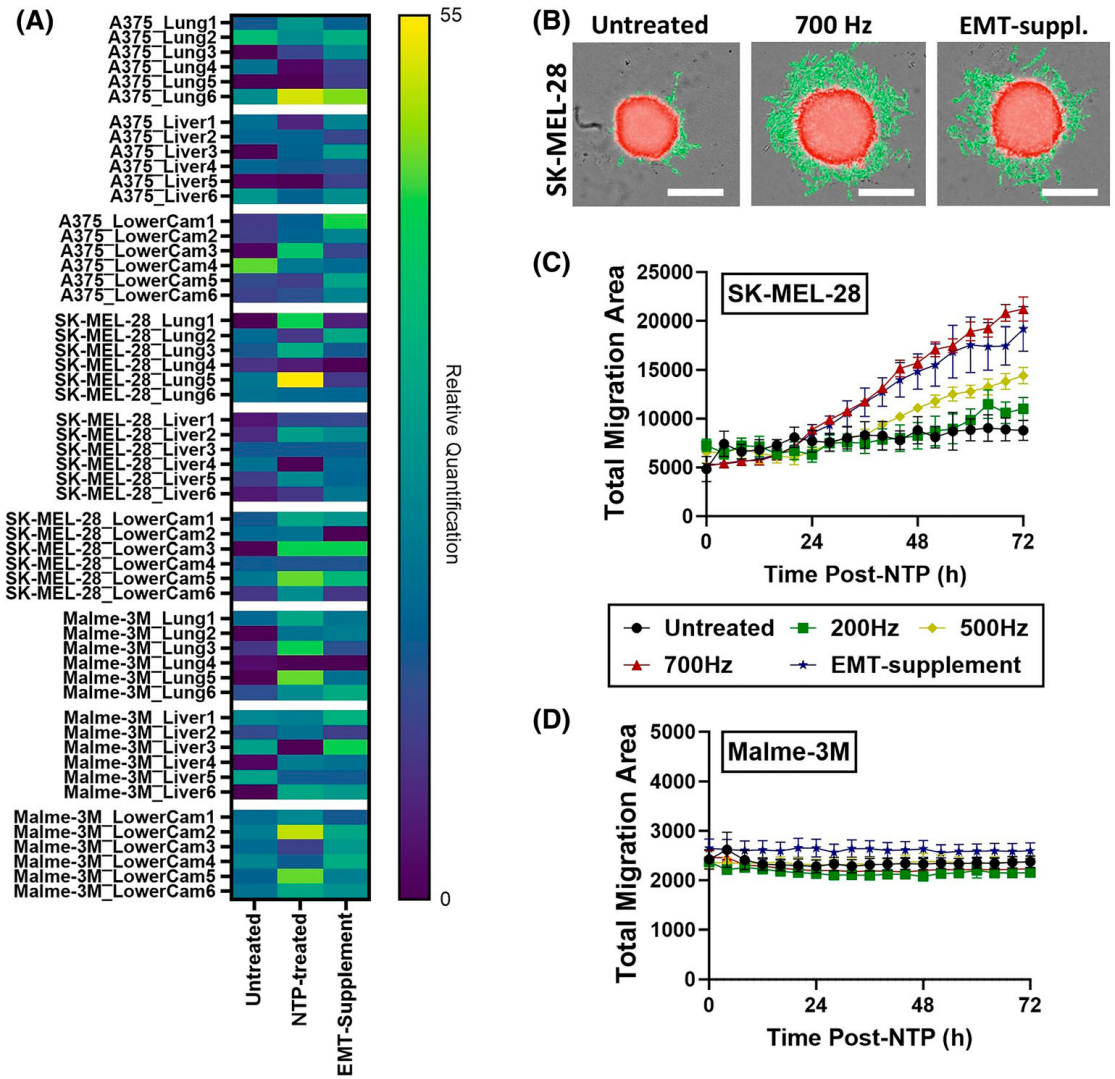
Functional assays to investigate enhanced migration post‐non‐thermal plasma (NTP) treatment. (A) Heatmap showing the presence of human RNA in different organs of the chicken embryo, indicating metastasis from the primary tumor site. A generalized linear mixed model was used and revealed no statistical difference in human RNA between the different conditions. (B) Representation of the analysis for the 3D spheroid migration software, analyzed by the orbits analysis software. The red masking represents the spheroid and the green masking represents the migration area out of the spheroid. Images were taken at 4× on the Tecan Spark Cyto 600. Scale bar represents 500 μm. (C, D) Results from the 3D spheroid migration assay for (C) SK‐MEL‐28 and (D) Malme‐3M. As a positive control, an epithelial‐mesenchymal transition (EMT)‐inducing supplement was used. No statistical tests were performed for this kinetic experiment, as the differences were negligible. EMT, epithelial‐mesenchymal transition; NTP, non‐thermal plasma. Data are represented as mean ± SEM of three independent experiments with 3–5 replicates per experiment.

In total, we collected samples from six eggs per condition for each cell line. Our results indicated that even for untreated tumors, human RNA was present in most of the samples (Fig. 4A), indicating the high migratory capacity of the melanoma cells. Due to large variations in the replicates, no statistical differences were found; though based on the heatmap (Fig. 4A), several trends in all three cell lines were observed. For instance, in the samples from the lung and lower CAM, more human RNA appeared to be detected in the NTP‐treated samples compared to the untreated samples. It also appeared that liver samples contained less human RNA compared to lung and lower CAM samples for all three cell lines. Overall, these data illustrated that the melanoma cells were able to migrate away from the primary tumor on top of the CAM and into the chicken embryo. Furthermore, while not statistically significant, it appeared that NTP‐treated melanoma tumors exhibit enhanced migratory capacity, which supported our molecular findings (Fig. 2).

To examine the migratory capacity in the 3D spheroid model, we performed a 3D migration assay by transferring the spheroids to collagen‐coated plates immediately after NTP treatment, following which they were monitored with live‐cell imaging every 4 h for 3 days. Analysis was performed using the Orbits Oncology analysis software, which recognized and calculated the area of migrating tumor cells (Fig. 4B) [36]. For the SK‐MEL‐28 spheroids, a substantial increase in migration was observed for the high‐dose NTP treatment, comparable to that of the positive control, EMT‐supplement (Fig. 4C). Interestingly, the low‐dose NTP‐treated spheroids did not show an increase in migration, which is in line with our molecular findings (Fig. 3). Neither NTP treatments nor the positive control induced alteration in Malme‐3M spheroids migration (Fig. 4D). Given that the 3D migration assay requires several transfer steps, it is plausible that the experiment induced substantial stress on the Malme‐3M spheroids, which are relatively less compact compared to SK‐MEL‐28 spheroids. Therefore, we were cautious in our assessment of EMT induction in these spheroids.

Taken together, these functional findings support in part the molecular evaluation of NTP‐induced EMT response. Furthermore, the dose‐dependent effects of NTP are also supported, as high‐dose NTP treatment enhanced the migratory capabilities of SK‐MEL‐28 spheroids, whereas the low‐dose NTP treatment did not.

## 4 Discussion

Although oxidative stress‐inducing therapies, like NTP, hold significant promise as cancer therapies, the dual nature of oxidative stress is often disregarded. Indeed, the majority of studies exploring NTP as a novel therapy for cancer report notable results in terms of cytotoxicity, selectivity, and immune‐stimulatory efficacy, whereas almost no studies investigate its potential to induce EMT and, consequently, its influence on the migratory properties of the tumor cells. In this study, we evaluated the expression of six key EMT biomarkers in melanoma cancer cells, using both the CAM *in ovo* model and the 3D spheroid model. To further evaluate our findings, a functional assay was performed for both 3D models, assessing the enhanced migratory capabilities of the cancer cells post‐NTP treatment. Taken together, our study demonstrated that NTP initiates an EMT‐inducing response for the high‐dose NTP treatment, whereas the low‐dose NTP did not affect the EMT status or migratory capabilities of the cells, yet still induced an anti‐cancer response.

The CAM model is very useful as a relatively simple and cost‐efficient approach to studying angiogenesis and metastasis. Because of the highly vascularized nature of the CAM, tumor grafting is relatively easy with high reproducibility and, moreover, it mimics the physiological cancer cell environment due to the presence of extracellular matrix proteins like fibronectin, laminin, collagen type I, and integrin α_V_β_3_ [41, 42]. While the use of this model dates back to studies from 1949, its re‐emergence as an inventive alternative to murine models has sparked renewed attention within the cancer research community [37]. We used this model for the formation of tumors for three melanoma cell lines, A375, SK‐MEL‐28, and Malme‐3M, and assessed key EMT biomarkers, as well as the presence of metastasis in the chicken embryo. Our results indicate that EMT is initiated after NTP treatment, as evidenced by activation of the cadherin switch, upregulation of vimentin, and upregulation of the three major EMT transcription factors (Fig. 1). These results are further supported by an elevated trend of metastasis in the lungs and lower CAM of the chicken embryo (Fig. 3). While the outcomes from this model undeniably establish the initiation of EMT, drawing conclusions about the progression of this process remains limited since these findings are only representative of one specific time point following NTP treatment.

Examination at different time points beyond day 14 with the CAM model is necessary but would require further regulatory approval, thus limiting the advantages of this model. Therefore, to look at later effects concerning the progression of NTP‐induced EMT, we used the 3D spheroid model, which does not have any regulatory limitations. In this model, we compared two NTP doses (200 Hz vs. 700 Hz) and examined the responses at distinct time points following NTP treatment (day 1, day 4, and day 7). We demonstrated that the low NTP treatment (at 200 Hz, 3.52 J) had a negligible impact on EMT initiation. This was observed not only through the evaluation of the EMT biomarkers but also in the assessment of migration after treatment (Figs 2 and 3). In contrast, the higher dose 700 Hz treatment (12.3 J) triggered an EMT response, as evidenced by the activation of all six key biomarkers in both cell lines, as well as enhanced migratory characteristics after treatment, but only for SK‐MEL‐28 (Figs 2 and 3). This can potentially be explained by the relative instability of the Malme‐3M spheroids in comparison to the SK‐MEL‐28 spheroids. Given that the 3D migration assay requires quite a lot of transferring of the spheroids, it is plausible that the experiment itself induced substantial stress on the spheroids. Therefore, we are cautious with any claims on EMT induction in these spheroids.

Alterations in the EMT biomarkers appeared to be temporary and most of the biomarkers returned to their baseline expression by day 7, implying that the cells might only go through partial EMT instead of reaching a fully mesenchymal phenotype. Nevertheless, it should be noted that these are the results from a single treatment. The effect of subjecting cells and tumors to repetitive NTP treatments should be evaluated, as it is probable that more sustained EMT stimulation might drive the cells to transition further and attain a fully mesenchymal phenotype. Therefore, it is clear that choosing the most optimal NTP treatment conditions is of great importance, particularly when translating this technology into the clinic. Whereas higher NTP treatment doses induce more cell death, the potential to simultaneously trigger adverse effects, like enhanced metastatic potential, cannot be ignored. In contrast, using lower NTP doses might lead to fewer side effects, though lower toxicity may require repetitive treatments. While this is not a significant concern for superficial tumors, like melanoma, other skin cancers, or head‐and‐neck cancer, it might be an important limitation for the treatment of tumors within the body, which require surgery to access the tumor site.

Given that the induction of EMT is a relatively common side effect in conventional therapies like chemotherapy and radiotherapy, increasing efforts are being made to search for opportunities to interfere with the EMT pathway and develop anti‐EMT adjuvant therapies [17, 43]. However, this is very challenging due to the high plasticity and heterogeneity of the various pathways involved [23]. Most of the currently available anti‐EMT drugs are focused on blocking upstream inducers of EMT such as TGF‐β [20, 44]. The majority of these blockers are broad‐spectrum inhibitors, being non‐specific to cancer cells, and therefore often cause side effects [20]. An alternative anti‐EMT approach is targeting the EMT transcription factors [20, 44]. A study showed that genetically silencing the transcription factors could reduce the metastatic burden and resistance to chemotherapy in a pancreatic cancer mouse model, demonstrating their potential as valuable targets [39, 45]. However, some controversy exists due to the traditional classification of transcription factors as ‘undruggable’ as it is a nuclear event which is not readily accessible for drugs [44, 46]. Another strategy is to target active biomarkers of EMT [44, 46]. For example, biologically active compounds against vimentin, fibronectin, and N‐cadherin could be used to promote the transition back into an epithelial phenotype. Although this appears very promising, defining the precise therapeutic window for this type of drug is critical as it might also accelerate the metastasis of disseminated cells [44]. Despite the many challenges of developing anti‐EMT therapies, there are currently several promising drugs at various stages of clinical trials, capable of targeting EMT markers. Trabedersen, an antisense therapy capable of blocking protein translation of TGF‐β mRNA, showed encouraging preliminary results in patients with stage IV pancreatic cancer, malignant melanoma, colorectal cancer, and brain cancer [46, 47]. Salinomycin, an ionophore antibiotic, has also shown promise, as it downregulated vimentin, upregulated E‐cadherin in colorectal cancer cells, reversed doxorubicin‐induced EMT, and restored chemosensitivity *in vitro* and *in vivo* [48]. Moreover, several clinical cases showed reduced tumor metastasis in patients with metastatic breast cancer, metastatic ovarian cancer, and metastatic head‐and‐neck squamous cell carcinoma [49].

In summary, despite notable progress, targeting or suppressing EMT remains very challenging. Developing anti‐cancer therapies that do not alter the EMT state of the cancer cells offers therefore major benefits. As shown by our results, optimizing the treatment parameters of NTP therapy could majorly impact its response on EMT induction, thereby highlighting the clinical potential of using lower NTP treatment doses.

## 5 Conclusions

While NTP holds remarkable potential as an anti‐cancer therapy, the potential to have adverse effects must also be evaluated and should not be shied away from. There is a major gap in knowledge on the capacity of NTP treatment to induce EMT and, subsequently, impact cell migration. This study provides a molecular and functional overview of NTP‐induced EMT initiation and progression in melanoma by evaluating the expression of six key EMT biomarkers together with changes in migration. We revealed that a high‐dose NTP treatment triggered a significant EMT response and enhanced migration following treatment, whereas low‐dose NTP did not alter the EMT state of the cells. Overall, our findings provide further insight into critical NTP‐cell interactions and highlight that optimization of NTP dose is key to improving the therapeutic potential for clinical translation of NTP.

## Conflict of interest

The authors declare no conflict of interest.

## Author contributions

Conception (EB, AL), experimental design (EB, AL, AP‐M), data acquisition (EB, HV, JDB, HWL), data analysis (EB, JDB, AL), data interpretation (EB, AL), creation of software (ECDLH), provided resources and supervision (WVB, SV, ES, AB, AL), drafted manuscript (EB, AL), and revised manuscript (EB, ECDLH, HV, JDB, HWL, AP‐M, WVB, SV, ES, AB, AL).

## Data Availability

The data that support the findings of this study are available from the corresponding author, abraham.lin@uantwerpen.be, upon reasonable request.

## References

[1] Graves DB . Low temperature plasma biomedicine: a tutorial review. Phys Plasmas. 2014;21(8):080901. 10.1063/1.4892534

[2] Keidar M . Plasma for cancer treatment. Plasma Sources Sci Technol. 2015;24(3):033001. 10.1088/0963-0252/24/3/033001

[3] Faramarzi F , Zafari P , Alimohammadi M , Moonesi M , Rafiei A , Bekeschus S . Cold physical plasma in cancer therapy: mechanisms, signaling, and immunity. Oxid Med Cell Longev. 2021;2021:9916796. 10.1155/2021/9916796 35284036 PMC8906949

[4] Živanić M , Espona‐Noguera A , Lin A , Canal C . Current state of cold atmospheric plasma and cancer‐immunity cycle: therapeutic relevance and overcoming clinical limitations using hydrogels. Adv Sci. 2023;10(8):e2205803. 10.1002/advs.202205803 PMC10015903 36670068

[5] Huang J , Chen W , Li H , Wang XQ , Lv GH , Khohsa ML , et al. Deactivation of A549 cancer cells in vitro by a dielectric barrier discharge plasma needle. J Appl Phys. 2011;109(5):053305‐053305‐6. 10.1063/1.3553873

[6] Kim SJ , Chung TH , Bae SH , Leem SH . Induction of apoptosis in human breast cancer cells by a pulsed atmospheric pressure plasma jet. Appl Phys Lett. 2010;97(2):023702‐023702‐3. 10.1063/1.3462293

[7] Hattori N , Yamada S , Tori K , Torii K , Takeda S , Nakamura K , et al. Effectiveness of plasma treatment on pancreatic cancer cells. Int J Oncol. 2015;47(5):1655–1662. 10.3892/ijo.2015.3149 26351772 PMC4599185

[8] Tanaka H , Mizuno M , Ishikawa K , Nakamura K , Kajiyama H , Kano H , et al. Plasma‐activated medium selectively kills glioblastoma brain tumor cells by Down‐regulating a survival signaling molecule, AKT kinase. Plasma Med. 2011;1(3–4):265–277. 10.1615/PlasmaMed.2012006275

[9] Leduc M , Guay D , Leask RL , Coulombe S . Cell permeabilization using a non‐thermal plasma. New J Phys. 2009;11:115021. 10.1088/1367-2630/11/11/115021

[10] Vermeylen S , De Waele J , Vanuytsel S , De Backer J , Van der Paal J , Ramakers M , et al. Cold atmospheric plasma treatment of melanoma and glioblastoma cancer cells. Plasma Processes Polym. 2016;13(12):1195–1205. 10.1002/ppap.201600116

[11] Vandamme M , Robert E , Dozias S , Sobilo J , Lerondel S , Le Pape A , et al. Response of human glioma U87 xenografted on mice to non thermal plasma treatment. Plasma Med. 2011;1(1):27–43. 10.1615/PlasmaMed.v1.i1.30

[12] Brullé L , Vandamme M , Riès D , Martel E , Robert E , Lerondel S , et al. Effects of a non thermal plasma treatment alone or in combination with gemcitabine in a MIA PaCa2‐luc orthotopic pancreatic carcinoma model. PLoS One. 2012;7(12):e52653. 10.1371/journal.pone.0052653 23300736 PMC3530450

[13] Lin A , De Backer J , Quatannens D , Cuypers B , Verswyvel H , De La Hoz EC , et al. The effect of local non‐thermal plasma therapy on the cancer‐immunity cycle in a melanoma mouse model. Bioeng Transl Med. 2022;7(3):e10314. 10.1002/btm2.10314 36176603 PMC9472020

[14] Troitskaya O , Golubitskaya E , Biryukov M , Varlamov M , Gugin P , Milakhina E , et al. Non‐thermal plasma application in tumor‐bearing mice induces increase of serum hmgb1. Int J Mol Sci. 2020;21(14):5128. 10.3390/ijms21145128 32698492 PMC7404183

[15] Wende K , von Woedtke T , Weltmann KD , Bekeschus S . Chemistry and biochemistry of cold physical plasma derived reactive species in liquids. Biol Chem. 2019;400:19–38. 10.1515/hsz-2018-0242 30403650

[16] Lin A , Gorbanev Y , De Backer J , Van Loenhout J , Van Boxem W , Lemière F , et al. Non‐thermal plasma as a unique delivery system of short‐lived reactive oxygen and nitrogen species for immunogenic cell death in melanoma cells. Adv Sci. 2019;6(6):1802062. 10.1002/advs.201802062 PMC6425452 30937272

[17] Giannoni E , Parri M , Chiarugi P . EMT and oxidative stress: a bidirectional interplay affecting tumor malignancy. Antioxid Redox Signal. 2012;16(11):1248–1263. 10.1089/ars.2011.4280 21929373

[18] Chatterjee R , Chatterjee J . ROS and oncogenesis with special reference to EMT and stemness. Eur J Cell Biol. 2020;99(2–3):151073. 10.1016/j.ejcb.2020.151073 32201025

[19] Ribatti D , Tamma R , Annese T . Epithelial‐mesenchymal transition in cancer: a historical overview. Transl Oncol. 2020;13(6):100773. 10.1016/j.tranon.2020.100773 32334405 PMC7182759

[20] Huang Y , Hong W , Wei X . The molecular mechanisms and therapeutic strategies of EMT in tumor progression and metastasis. J Hematol Oncol. 2022;15(1):129. 10.1186/s13045-022-01347-8 36076302 PMC9461252

[21] Pastushenko I , Blanpain C . EMT transition states during tumor progression and metastasis. Trends Cell Biol. 2019;29(3):212–226. 10.1016/j.tcb.2018.12.001 30594349

[22] Yang J , Antin P , Berx G , Blanpain C , Brabletz T , Bronner M , et al. Guidelines and definitions for research on epithelial–mesenchymal transition. Nat Rev Mol Cell Biol. 2020;21(6):341–352. 10.1038/s41580-020-0237-9 32300252 PMC7250738

[23] Ye X , Weinberg RA . Epithelial‐mesenchymal plasticity: a central regulator of cancer progression. Trends Cell Biol. 2015;25(11):675–686. 10.1016/j.tcb.2015.07.012 26437589 PMC4628843

[24] Sinha D , Saha P , Samanta A , Bishayee A . Emerging concepts of hybrid epithelial‐to‐mesenchymal transition in cancer progression. Biomolecules. 2020;10(11):1561. 10.3390/biom10111561 33207810 PMC7697085

[25] Jolly MK , Mani SA , Levine H . Hybrid epithelial/mesenchymal phenotype(s): the ‘fittest’ for metastasis? Biochim Biophys Acta Rev Cancer. 2018;1870(2):151–157. 10.1016/j.bbcan.2018.07.001 29997040

[26] Yehl M , Kucharski D , Eubank M , Gulledge B , Rayan G , Uddin MG , et al. The development of nonthermal plasma and Tirapazamine as a novel combination therapy to treat melanoma in situ. Cells. 2023;12(16):2113. 10.3390/cells12162113 37626923 PMC10453358

[27] Friedman PC , Miller V , Fridman G , Lin A , Fridman A . Successful treatment of actinic keratoses using nonthermal atmospheric pressure plasma: a case series. J Am Acad Dermatol. 2017;76(2):349–350. 10.1016/j.jaad.2016.09.004 28088998

[28] Pasqual‐Melo G , Gandhirajan RK , Stoffels I , Bekeschus S . Targeting malignant melanoma with physical plasmas. Clin Plasma Med. 2018;10:1–8. 10.1016/j.cpme.2018.03.001

[29] Chu PY , Koh APF , Antony J , Huang RYJ . Applications of the Chick Chorioallantoic membrane as an alternative model for cancer studies. Cells Tissues Organs. 2022;211(2):222–237. 10.1159/000513039 33780951 PMC9153341

[30] Nowak‐Sliwinska P , Segura T , Iruela‐Arispe ML . The chicken chorioallantoic membrane model in biology, medicine and bioengineering. Angiogenesis. 2014;17(4):779–804. 10.1007/s10456-014-9440-7 25138280 PMC4583126

[31] Duval K , Grover H , Han LH , Mou Y , Pegoraro AF , Fredberg J , et al. Modeling physiological events in 2D vs. 3D cell culture. Phys Ther. 2017;32(4):266–277. 10.1152/physiol.00036.2016 PMC5545611 28615311

[32] Melissaridou S , Wiechec E , Magan M , Jain MV , Chung MK , Farnebo L , et al. The effect of 2D and 3D cell cultures on treatment response, EMT profile and stem cell features in head and neck cancer. Cancer Cell Int. 2019;19(1):16. 10.1186/s12935-019-0733-1 30651721 PMC6332598

[33] Lin A , Gromov M , Nikiforov A , Smits E , Bogaerts A . Characterization of non‐thermal dielectric barrier discharges for plasma medicine: from plastic well plates to skin surfaces. Plasma Chem Plasma Proc. 2023;43(6):1587–1612. 10.1007/s11090-023-10389-w

[34] Lin A , Biscop E , Gorbanev Y , Smits E , Bogaerts A . Toward defining plasma treatment dose: the role of plasma treatment energy of pulsed‐dielectric barrier discharge in dictating in vitro biological responses. Plasma Processes Polym. 2022;19(3):151. 10.1002/ppap.202100151

[35] Terrones M , Deben C , Rodrigues‐Fortes F , Schepers A , de Beeck KO , van Camp G , et al. CRISPR/Cas9‐edited ROS1+ non‐small cell lung cancer cell lines highlight differential drug sensitivity in 2D vs 3D cultures while reflecting established resistance profiles. J Transl Med. 2024;22(1):234. 10.1186/s12967-024-04988-0 38433235 PMC10910754

[36] Deben C , De La Hoz EC , Le Compte M , Van Schil P , Hendriks JMH , Lauwers P , et al. OrBITS: label‐free and time‐lapse monitoring of patient derived organoids for advanced drug screening. Cell Oncol. 2023;46(2):299–314. 10.1007/s13402-022-00750-0 PMC10060271 36508089

[37] Ribatti D . The Chick embryo Chorioallantoic membrane in the study of angiogenesis and metastasis. Angiogenesis. 2010;11:311–319. 10.1007/s10456-008-9117-1 18780151

[38] Loh CY , Chai JY , Tang TF , Wong W , Sethi G , Shanmugam M , et al. The e‐cadherin and n‐cadherin switch in epithelial‐to‐mesenchymal transition: signaling, therapeutic implications, and challenges. Cells. 2019;8(10):1118. 10.3390/cells8101118 31547193 PMC6830116

[39] Debnath P , Huirem RS , Dutta P , Palchaudhuri S . Epithelial‐mesenchymal transition and its transcription factors. Biosci Rep. 2022;42(1):BSR20211754. 10.1042/BSR20211754 34708244 PMC8703024

[40] Jägle S , Dertmann A , Schrempp M , Hecht A . ZEB1 is neither sufficient nor required for epithelial‐mesenchymal transition in LS174T colorectal cancer cells. Biochem Biophys Res Commun. 2017;482(4):1226–1232. 10.1016/j.bbrc.2016.12.017 27923654

[41] Ribatti D . The CAM assay in the study of the metastatic process. Exp Cell Res. 2021;400(2):112510. 10.1016/j.yexcr.2021.112510 33524363

[42] Pawlikowska P , Tayoun T , Oulhen M , Faugeroux V , Rouffiac V , Aberlenc A , et al. Exploitation of the chick embryo chorioallantoic membrane (CAM) as a platform for anti‐metastatic drug testing. Sci Rep. 2020;10(1):16876. 10.1038/s41598-020-73632-w 33037240 PMC7547099

[43] Santos Ramos F , Wons L , João Cavalli I , Ribeiro E . Epithelial‐mesenchymal transition in cancer: an overview. Integr Cancer Sci Ther. 2017;4(3):243. 10.15761/icst.1000243

[44] Jonckheere S , Adams J , De Groote D , Campbell K , Berx G , Goossens S . Epithelial‐mesenchymal transition (EMT) as a therapeutic target. Cells Tissues Organs. 2022;211(2):157–182. 10.1159/000512218 33401271

[45] Sakata J , Utsumi F , Suzuki S , Niimi K , Yamamoto E , Shibata K , et al. Inhibition of ZEB1 leads to inversion of metastatic characteristics and restoration of paclitaxel sensitivity of chronic Chemoresistant ovarian carcinoma cells. Oncotarget. 2017;8(59):99482–99494. 10.18632/oncotarget.20107 29245917 PMC5725108

[46] Zhang N , Ng AS , Cai S , Li Q , Yang L , Kerr D . Novel therapeutic strategies: targeting epithelial–mesenchymal transition in colorectal cancer. Lancet Oncol. 2021;22(8):e358–e368. 10.1016/S1470-2045(21)00343-0 34339656

[47] D'Cruz O , Lee C , Trieu V , Hwang L . Synergistic antitumor effects of OT‐101 (trabedersen), a transforming growth factor‐beta 2 (TGF‐β2) antisense oligonucleotide (ASO) and chemotherapy in preclinical tumor models. Ann Oncol. 2017;28:v583–v584. 10.1093/annonc/mdx390.034

[48] Zhou Y , Liang C , Xue F , Chen W , Zhi X , Feng X , et al. Salinomycin decreases doxorubicin resistance in hepatocellular carcinoma cells by inhibiting the β‐catenin/TCF complex association via FOXO3a activation. Oncotarget. 2015;6(12):10350–10365. 10.18632/oncotarget.3585 25871400 PMC4496360

[49] Qi D , Liu Y , Li J , Huang JH , Hu X , Wu E . Salinomycin as a potent anticancer stem cell agent: state of the art and future directions. Med Res Rev. 2022;42(3):1037–1063. 10.1002/med.21870 34786735 PMC9298915

